# Draft genome sequence of a multidrug-resistant clinical *Serratia marcescens* DUEML4 (ST444) isolated from a human tracheal aspirate in Bangladesh

**DOI:** 10.1128/mra.00277-25

**Published:** 2025-04-29

**Authors:** Tasnimul Arabi Anik, Faruk Islam, Shahin Ara Begum, Humaira Akhter, Anowara Begum

**Affiliations:** 1 Environmental Microbiology Laboratory, Department of Microbiology, University of Dhaka 95324, Dhaka, Dhaka Division, Bangladesh; 2 Laboratory Medicine, Green Life Hospital Ltd 468842 , Dhaka, Bangladesh; Rochester Institute of Technology, Rochester, New York, USA

**Keywords:** *Serratia marcescens*, respiratory tract infection, genome sequence, antimicrobial resistance

## Abstract

*Serratia marcescens* is a rare opportunistic nosocomial pathogen. Here, we report the draft genome sequence of *S. marcescens* DUEML4 (ST444), isolated from the tracheal aspirate of a 68-year-old female patient with respiratory tract infection in a hospital in Dhaka. This is the first public genome sequence of ST444.

## ANNOUNCEMENT

According to WHO, lower respiratory tract infections (LRTIs) have been a leading cause of death in low-income countries (1). *Serratia marcescens* is a rare opportunistic nosocomial pathogen with the ability to cause respiratory tract infection, particularly in immunocompromised individuals (2). Here, we report *S. marcescens* DUEML4, a multidrug-resistant bacterium isolated from the tracheal aspirate of a 68-year-old female patient with LRTI in a hospital in Dhaka (23°44′46.8″N; 90°23′9.1″E). The sample was collected by a trained medical professional using a sterile suction catheter. The sample was vortexed, serially diluted in PBS, and plated onto MacConkey and blood agar. After aerobic incubation at 37°C for 18–24 hours, DUEML4 appeared as pale, non-lactose fermenting colonies on MacConkey agar and smooth, pigmented (prodigiosin) colonies on blood agar at 30°C. Isolate was confirmed via biochemical tests (TSI, MIU, citrate, catalase, oxidase) and 16S rRNA sequencing. Strain DUEML4 was resistant to all tested antibiotics, including carbapenems (imipenem, meropenem), tetracyclines (tetracycline, doxycycline), aminoglycosides (gentamicin, amikacin), fluoroquinolones (ciprofloxacin, levofloxacin), cephems (ceftazidime, cefepime, cefotaxime), β-lactam combination agent (piperacillin-tazobactam), trimethoprim-sulfamethoxazole, and colistin according to the CLSI M100-Ed35:2025 guidelines. Due to the limited genomic data on LRTI-associated *S. marcescens*, we sequenced and analyzed the genome to better understand its resistance and virulence.

A single colony was cultured in LB broth with overnight incubation at 37°C, and genomic DNA was extracted using the QIAamp DNA Mini Kit (QIAGEN) following the manufacturer’s protocol. NGS library preparation was performed using the Illumina DNA Prep kit (Illumina, San Diego, CA, USA), and sequencing was carried out on the Illumina NextSeq 550 platform. A total of 725,031 raw reads were generated, corresponding to 347.3 Mbp of sequencing data (read length 250 bp). The sequenced genome had an average depth of coverage of 47.17×. In the sequence analysis, default parameters were used. Raw reads were quality-checked using FastQC (v0.12) and trimmed with Trimmomatic (v0.39) (3, 4). Assembly was done using SPAdes (v4.0.0), and species identification was confirmed via KmerFinder (v3.0.2) with 95.59% ANI to *S. marcescens* ATCC 13880 (GCA_017301275.1) (5, 6). Annotation of the genome was performed by Prokka (v1.14.5) and the NCBI PGAP (7, 8). Antimicrobial resistance genes were identified using ResFinder v3.1, NCBI v4.0.19, CARD v3.2.4, and MEGARes v3.0 (9–12). Virulence genes were identified using the VFDB v2.0 (13). The sequence type (ST) was identified using PubMLST (https://pubmlst.org/) (14).

The genome assembly (13 contigs, N50: 2.9 Mb) had 95.72% completeness and 3.49% contamination per CheckM (v1.2.3) (15). Afterward, MLST analysis identified the isolate as ST444. The assembled genome was 5.1 Mb in length (60% GC content). No plasmid was detected. A total of 4,845 genes were identified, including 4,725 protein coding and 93 RNA coding. Eight resistance determinants, including *blaSRT-1*, *oqxB9*, *aac(6’)-Ic, mexI, sdeB, sdeX, sdeY,* and *hns,* were detected (Table 1). Seven insertion sequences from four families (IS3, ISL3, Tn3, and IS481) were detected by ISfinder (accessed on 11 February 2025), and PathogenFinder (v1.1) predicted an 86.8% probability of pathogenicity. This genomic analysis highlights the potential role of ST444 as a human pathogen, emphasizing its multidrug resistance profile.

**TABLE 1:** Antimicrobial resistance and virulence genes in *S. marcescens* DUEML4

Database	Gene	Function	Resistance
ncbi, resfinder, card, megares	*blaSRT-1*	SRT/SST family class C β-lactamase	Cephalosporins
ncbi, resfinder, card	*oqxB9*	multidrug efflux RND transporter permease subunit OqxB9	Phenicols, quinolones
ncbi, resfinder, card, megares	*aac(6')-Ic*	AAC(6')-Ic is a chromosomal-encoded aminoglycoside acetyltransferase in *S. marcescens*	Aminoglycosides
card, megares	*mexI*	MexI is the inner membrane transporter of the efflux complex MexGHI-OpmD.	Acridine dyes, fluoroquinolones, tetracyclines
card, megares	*hns*	H-NS is a histone-like protein involved in global gene regulation in Gram-negative bacteria. It is a repressor of the membrane fusion protein genes acrE, mdtE, and emrK as well as nearby genes of many RND-type multidrug exporters.	Cephalosporins, cephamycin, fluoroquinolones, macrolides, penam, tetracyclines
megares	*sdeB, sdeX, sdeY*	Multi-compound: Drug and biocide resistance: Drug and biocide RND efflux regulator	Variety of antibiotics
vfdb	*cheY*	(*cheY*) chemotaxis regulatory protein CheY [peritrichous flagella (AI145)] [*Yersinia enterocolitica* subsp. *enterocolitica* 8081]	Not applicable
vfdb	*fliM*	(*fliM*) flagellar motor switch protein FliM [Flagella (VF0394)] [*Yersinia enterocolitica* subsp. *enterocolitica* 8081]	Not applicable
vfdb	*fliG*	(*fliG*) flagellar motor switch protein G [Flagella (VF0394)] [*Yersinia enterocolitica* subsp. *enterocolitica* 8081]	Not applicable

## Data Availability

The raw reads can be found at NCBI Sequence Read Archive (SRA), accession number SRS23885608. The assembly of raw reads is available at NCBI BioProject, accession number PRJNA1215361 and BioSample accession number SAMN46402925. The complete genome sequence is available in GenBank under accession number JBLFED000000000.1.
